# Development of DNA Vaccine Candidate against SARS-CoV-2

**DOI:** 10.3390/v14051049

**Published:** 2022-05-15

**Authors:** Xingyun Wang, Nino Rcheulishvili, Jie Cai, Cong Liu, Fengfei Xie, Xing Hu, Nuo Yang, Mengqi Hou, Dimitri Papukashvili, Yunjiao He, Peng George Wang

**Affiliations:** Department of Pharmacology, School of Medicine, Southern University of Science and Technology, Shenzhen 518000, China; 12032609@mail.sustech.edu.cn (X.W.); nino@sustech.edu.cn (N.R.); caij@mail.sustech.edu.cn (J.C.); 11930759@mail.sustech.edu.cn (C.L.); xieff@mail.sustech.edu.cn (F.X.); 12032620@mail.sustech.edu.cn (X.H.); 12032625@mail.sustech.edu.cn (N.Y.); 11930141@mail.sustech.edu.cn (M.H.); dimitri@sustech.edu.cn (D.P.)

**Keywords:** DNA vaccine, COVID-19, SARS-CoV-2

## Abstract

Despite the existence of various types of vaccines and the involvement of the world’s leading pharmaceutical companies, severe acute respiratory syndrome coronavirus 2 (SARS-CoV-2) remains the most challenging health threat in this century. Along with the increased transmissibility, new strains continue to emerge leading to the need for more vaccines that would elicit protectiveness and safety against the new strains of the virus. Nucleic acid vaccines seem to be the most effective approach in case of a sudden outbreak of infection or the emergence of a new strain as it requires less time than any conventional vaccine development. Hence, in the current study, a DNA vaccine encoding the trimeric prefusion-stabilized ectodomain (S1+S2) of SARS-CoV-2 S-protein was designed by introducing six additional prolines mutation, termed HexaPro. The three-dose regimen of designed DNA vaccine immunization in mice demonstrated the generation of protective antibodies.

## 1. Introduction

Severe acute respiratory syndrome coronavirus 2 (SARS-CoV-2) represents number seven coronavirus that infects humans [1], causing the coronavirus disease (COVID-19). COVID-19 has been adversely impacting the world humanity since its emergence in 2019 [2,3]. Extremely high rate of transmissibility, disease severity, and immensely high frequency of newly arisen strains continue to have a huge direct [4] or indirect [5] impact on public health and economic losses [6]. Thus, this markedly impedes the control measures for the spread of SARS-CoV-2 [7]. All of these lead to the urgency of vaccines notwithstanding the number of vaccine candidates in or approaching the clinical studies and even already approved commercially available vaccines [8,9,10,11]. Major technological innovations and investments in research promoted the start of a new era in vaccinology with regard to the employment of nucleic acids for immunization. Indeed, currently, the most effective vaccines against SARS-CoV-2 are considered mRNA vaccines that showed the fastest development in medical history [12]. However, despite their advantages [13], DNA vaccines deserve no less attention. The key beneficial features of DNA vaccines include the following: DNA vaccines induce cellular and humoral immunity while they do not induce an anti-vector immune response, DNA vaccines are effective even in the new-born children, they are safe, stable, the manufacturing technology itself is simple, fast [14,15], and inexpensive [16]. DNA vaccines can be processed at 4 °C which makes them much more durable compared with the mRNA technology [17]. Additionally, the development and production of DNA vaccines take less time in contrast to mRNA vaccines [17]. Notably, spike (S) protein, which is a viral glycoprotein protruded on the surface of SARS-CoV-2, represents a major target for the design and development of vaccines as it is responsible for the attachment to the host cell surface via binding to the angiotensin-converting enzyme 2 (ACE2), mediating viral entry and, consequently, leading to an infection. Additionally, binding to the ACE2 receptor along with the furin cleavage at the boundary of S1 and S2 subunits induces S2-mediated fusion of viral and host membranes [18]. Hence, both subunits have their distinct functions in the progression of viral entry and infection. In vaccine development, for the prefusion-stabilization of S-protein six, proline substitution (HexaPro) is often performed as it elicits higher expression and resistance to various temperature conditions, which gives better stability [19,20]. Additionally, D614G mutation in S-protein demonstrates the augmented susceptibility of SARS-CoV-2 to neutralization [21]. Foldon tag– trimerization domain is often used for structure stabilization and increased immunogenicity [22,23,24]. In the current study, a DNA vaccine encoding the ectodomain of S-protein that includes receptor-binding domain (RBD) was designed as the candidate for the development of the anti-SARS-CoV-2 vaccine. The DNA vaccine final construct comprised the ectodomain (S1+S2) with 6P [19] and D614G [25] mutagenesis has been employed in this study, and a C-terminal domain of T4 fibritin–trimeric foldon tag was fused at the C-terminal [26]. The results of the immunization of the mice and the immune response were evaluated.

## 2. Materials and Methods

### 2.1. Design and Synthesis of SARS-CoV-2 DNA Vaccine Construct

The SARS-CoV-2 S-protein sequence of Wuhan isolate (Accession number: NC_045512) was retrieved from available SARS-CoV-2 full genome sequences published on NCBI. The ectodomain of S-protein S1+S2 (1-1213) with the IgE signal peptide at the N-terminal, a trimeric foldon tag at the C-terminal, and 7 points mutation (D614G and 6P– F817P, A892P, A899P, A942P, K986P, V987P) was used. His tag and trimeric tag at the C-terminus were added. The optimized DNA sequence was synthesized, digested with BamHI and XhoI, and cloned into the expression vector PCDNA3.1 under the control of the human cytomegalovirus immediate-early promoter and a bovine growth hormone polyadenylation signal. The plasmid was synthesized by GenScript (Nanjing, China). Hence, the final DNA vaccine construct was IgE-spike-S1/S2-D614G-6P-foldon.

### 2.2. Amplification of the Target Gene

The “IgE-spike-S1/S2-D614G-6P-foldon” plasmid was transformed into DH5α competent cells via the heat-shock method– ice bath for 30 min, heat in 42 °C water bath for 90 s, followed by the ice bath again for 5 min. For the selection of ampicillin-resistant bacterial cells, the bacteria were inoculated on a solid Luria-Bertani (LB) medium containing ampicillin and incubated at 37 °C for 16 h. The single colony was identified by polymerase chain reaction (PCR), and the correct bacterial colony was transferred into a 2.5 L conical flask, placed in a 37 °C incubator, and shaken at 250 r/min for 12 h. After the cultivation, the liquid medium was centrifuged and the bacterial pellet was stored in a −80 °C refrigerator. Plasmid extraction was performed on the obtained bacterial pellet according to the instructions of the plasmid DNA extraction kit (Vazyme, Nanjing, China).

### 2.3. In Vitro Validation of DNA Vaccine Expression

Human embryonic kidney cells, HEK-293T (ATCC^®^ CRL-3216™) were maintained in DMEM supplemented with 10% fetal bovine serum (FBS) and penicillin-streptomycin. Cells were transfected with pDNA using Lipofectamine 2000 (ThermoFisher Scientific, Massachusetts, USA) transfection reagent following the manufacturer’s protocol. Forty-eight hours later cells were harvested and lysed using a modified RIPA cell lysis buffer. Proteins were separated on a 4–20% BIS-TRIS gel (ThermoFisher Scientific, Massachusetts, USA), then following transfer, blots were incubated with an anti-his antibody with horseradish peroxidase (HRP)-conjugated (Proteintech, Rosemont, USA).

### 2.4. Mouse Immunization Experiment

Female, 6-week-old BALB/c mice were purchased from Kangde Biological (Guangzhou, China). All animal experiments and research comply with all relevant ethical regulations, and the research has been ethically approved by the Animal Experiment Center of Southern University of Science and Technology. The DNA vaccine was injected into mice on days 0, 14, and 28. PCDNA3.1-spike was diluted with PBS to 25, 100, 200, 400 μg/mL, each group comprised 4 mice. The volume of the intramuscular injection (i.m.) was 100 μL per mouse. The control group was treated in the same way with the empty vector. The skin was cut around the administration site for about 1 square centimeter and the electroporation was performed. The voltage was 80 V, the current—300 mA, and the pulse generator was implemented when the resistance was 600–800 kΩ according to the parameters. Retro-orbital blood collection (volume 100 μL) was carried out every 7 days after immunization.

### 2.5. Detection of Plasma IgG and Antibody Titers by ELISA

ELISAs (ELISA supplemental solution set, Cat:SEKCR02, SinoBiological, Beijing, China) were performed to determine sera antibody binding titers. ELISA plates were coated with 200 ng recombinant protein antigens in coating buffer overnight at 4 °C. Plates were washed three times and then blocked with blocking buffer for 2 h at 37 °C. Plates were then washed and incubated with serial dilutions of mouse sera and incubated for 2 h at 37 °C. Plates were again washed and then incubated with 1:10,000 dilution of (HRP) conjugated anti-mouse IgG secondary antibody (Abcam, Boston, MA, USA) and incubated for 1 h at room temperature (RT). Finally, plates were washed for the third time, TMB was used as a substrate for 10 min, and the reaction was terminated with the stop solution. A microplate reader was employed to measure the reading at 450 nm wavelength.

### 2.6. SARS-CoV-2 Surrogate Virus Neutralization ELISA

cPass^TM^SARS-CoV-2 neutralization antibody detection Kit, REF:L00847 (GenScript, Nanjing, China) was used for the detection of circulating neutralizing antibodies. Firstly, serial dilutions of mouse serum were mixed with recombinant RBD-HRP at 37 °C for 30 min. Then the mixtures were added into ACE2 pre-coated plate wells and incubated at 37 °C or 15 min. Ultimately, after a final wash of the plate, a 1-step TMB-ELISA substrate was used and the reaction was stopped with a stop solution. The absorbance was detected at 450 nm wavelength within 30 min using a microplate reader.

### 2.7. SARS-CoV-2 Pseudovirus Neutralization Assay

The production of vesicular stomatitis virus (VSV)-based SARS-CoV-2 pseudovirus was performed as described previously [27]. Briefly, the backbone of the pseudotyped virus comes from the VSV virus, in which the G gene is replaced with the firefly luciferase (Fluc) reporter gene, and the S-protein from SARS-CoV-2 is incorporated as the membrane protein on the surface of the VSV pseudotyped virus. For the neutralization assay, HEK293-ACE2 cells were seeded 20,000 cells per well in 96-well cell culture plates and incubated until 85–90% confluency. Serum samples were 3-fold diluted and mixed with pseudovirus at 37 °C for 1 h. The mixture was added to the seeded cells. After 36 h, the Fluc activity was obtained by using the Bio-Lite Luciferase Assay System (Vazyme, Nanjing, China). The percentage of neutralization was calculated and EC50 titers were determined. The data for calculation of EC50 values is given in Supplementary File S1.

### 2.8. Statistics

The statistical analyses were performed using one-way ANOVA or t-test with GraphPad Prism 8.01 software. Data were considered significant if the *p*-value was <0.05. The normality of data was tested and normal distribution was confirmed by the normality and log-normality tests of column analysis via Graphpad Prism. Lines in all graphs represent means and error bars represent standard deviations that were generated via performing three parallel measurements for each sample. No samples or animals were excluded from the analysis.

## 3. Results

### 3.1. Characterization of SARS-CoV-2 DNA Vaccine Candidate and Validation In Vitro

**Figure 1:**
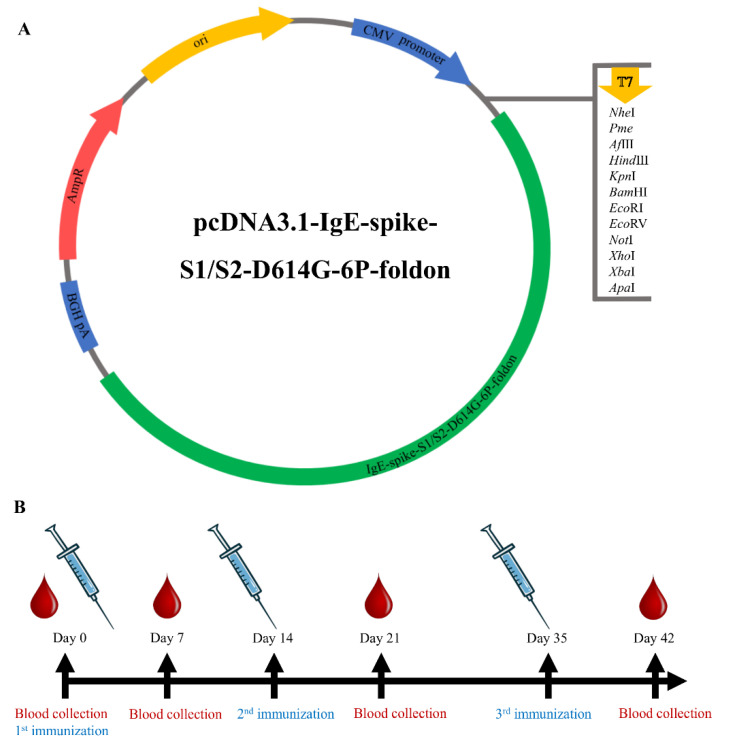
Vaccine construct and immunization timeline. (**A**) DNA construct encoding IgE-spike-S1/S2-D614G-6P-foldon. (**B**) Study scheme: timeline of immunization and blood collection.

**Figure 2:**
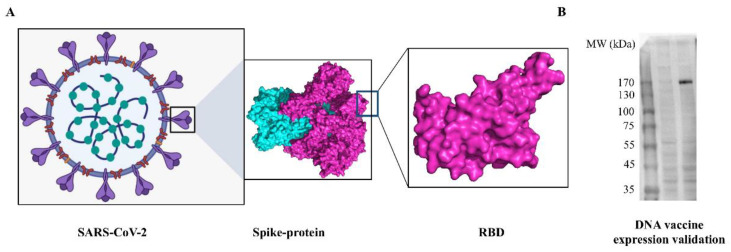
3D structure of S-protein ectodomain and expression validation of DNA vaccine expression. (**A**) 3D structure of S-protein ectodomain and RBD. (**B**) Validation of IgE-spike-S1/S2-D614G-6P-foldon expression in cell supernatant via western blotting.

DNA vaccine that encodes SARS-CoV-2 S-protein ectodomain was designed and optimized (Figure 1A). The complete sequence of DNA vaccine is given in Supplementary File S2. For the validation of antigen expression, the synthesized DNA construct was transfected into HEK-293T cells. As a result, a protein with a molecular weight of about 170 kDa was successfully expressed and secreted into the cell supernatant (Figure 2).

### 3.2. DNA Vaccine Candidate Induced Humoral Immune Response In Vivo

**Figure 3:**
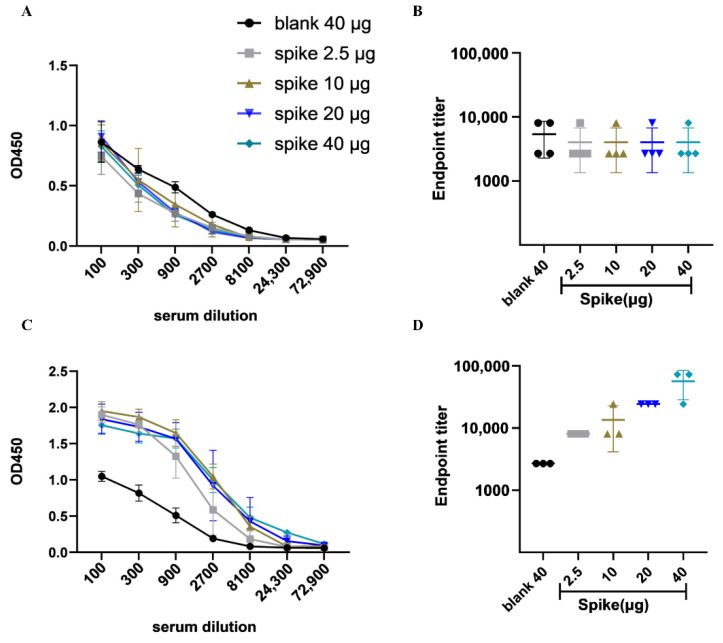
Humoral immune response to SARS-CoV-2 antigen in vaccinated BALB/c mice. (**A**) The antibody titer in sera of immunized mice after the first vaccination. (**B**) Endpoint titer in mice after the first immunization. (**C**) The antibody titer in mice after the third immunization. (**D**) Endpoint titer in mice after the third vaccination.

ELISA assay demonstrated that immunization with the DNA vaccine induced antibodies specific to SARS-CoV-2 S-protein after the booster dose (Figure 3C) in the blood sera collected on the 42nd day (Figure 1B). The antibody titers of different doses did not show an increase after the prime immunization (Figure 3A) compared with the control group, while a significantly increased concentration of IgG antibody was observed after the third immunization. As well, the endpoint titers (EPTs) showed consistent results (Figure 3B,D).

### 3.3. Inhibition of SARS-CoV-2 RBD Binding to a Host Receptor ACE2 via Competitive ELISA

**Figure 4:**
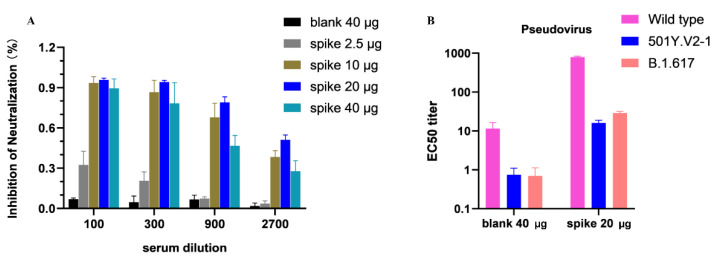
Humoral immune response in IgE-spike-S1/S2-D614G-6P-foldon-vaccinated mice. (**A**) Inhibition of SARS-CoV-2 S-protein binding to ACE2 receptor detected by ELISA. (**B**) Pseudovirus neutralization assay of the DNA-vaccine group shows the EC50 titers for the SARS-CoV-2 pseudovirus.

Detecting the antibodies that can inhibit the binding between the S-protein of SARS-CoV-2 and host receptor ACE2 is pivotal in the successful process of vaccine development against COVID-19. Thus, in this study, receptor binding inhibition capacity generated via the DNA vaccine-induced antibodies was measured. As a result, the sera collected from the three-dose-immunized mice inhibited the binding of SARS-CoV-2 S1/S2 ectodomain to ACE2 meaning that the vaccine-induced antibodies competed with the binding host receptor binding to RBD (Figure 4A).

### 3.4. Neutralization Ability DNA Vaccine-Induced Antibodies

The neutralization ability of the antibodies induced by the IgE-spike-S1/S2-D614G-6P-foldon vaccine against SARS-CoV-2 variants in vitro was evaluated. The pseudovirus neutralization assays were carried out using SARS-CoV-2 wild type (D614), 501Y.V2-1, and B.1.617 pseudoviruses. The results demonstrated that the sera obtained from the vaccinated mice could neutralize wild type SARS-CoV-2 with higher neutralizing activity (Figure 4B).

## 4. Discussion

The world’s leading pharmaceutical companies have initiated working on vaccine development since the genome sequence of SARS-CoV-2 became public on 11 January 2020 [28]. Currently, many types of vaccines are in use, in clinical, or preclinical development. Commercially available vaccines include protein-based, inactivated viruses, non-replicating viral vectors, and RNA-based vaccines. Among all of the mentioned vaccines mRNA vaccines elicit the most effectiveness while all of the vaccines appear to be protective and safe [29,30]. Indeed, currently authorized mRNA vaccines for emergency use that are BNT162b2 developed by BioNTech/Pfizer and mRNA-1273 by Moderna elicit 95% and 94.5% infection prevention effectiveness, respectively [31]. As to the non-mRNA-based vaccines, AstraZeneca has announced 70%, while Sinopharm elicited 79% efficacy [31]. Besides, there are a huge number of the whole virion-, viral vector-, nucleic acid-, and recombinant protein-based COVID-19 vaccines in phase 1–4 clinical trials [32]. Nevertheless, despite the presence and availability of vaccines against SARS-CoV-2 [9,33], the need for more, highly effective and safe vaccines remains still emergent to finally combat this infectious disease. There was no nucleic acid-based vaccine available until the COVID-19 pandemic emerged. The mRNA-based vaccine has already proved its high efficacy against SARS-CoV-2 [30]. A number of mRNA vaccines against other infectious diseases is in clinical development [34]. Indeed, the mRNA-LNP platform has been demonstrated to be a simple and effective approach in designing, developing, and producing the vaccine in a very short time [34,35]. However, the features of the DNA vaccine unquestionably deserve to be considered in the development of future vaccines. Both mRNA and DNA vaccines carry the genetic information to the host cells to instruct them to make antigens that are proteins similar to the virus [36,37]. The immune system of the host body responds to the immunization and the body is prepared for the fight with the real virus in case of infection [36,37]. mRNA needs to be delivered only in the cytoplasm where it can be directly translated into the desired antigen proteins while DNA needs to be transported into the nucleus first, where it will be transcribed and then translocated into the cytoplasm where the translation will take place [38]. However, despite its entry into the nucleus, according to the evidence, the possibility of genome integration in case of DNA vaccine administration is extremely low [15]. This is also consolidated by the fact that there is a number of clinical trials of prophylactic DNA vaccines for the prevention of various infectious diseases (NCT04591184, NCT01487876, NCT04445389, NCT01498718, etc.). The technique of developing an mRNA-based vaccine from the beginning to the step of commercialization comprises antigen selection, optimization (addition of immunogenic sequences), plasmid design and synthesis, plasmid transformation into competent bacterial cells, its amplification, extraction, purification, linearization, in vitro transcription of mRNA, purification, capping, packing of mRNA in an effective delivery system, in vitro validation of the antigen expression, in vivo analysis, and ultimately, clinical studies [39,40]. Notably, the DNA vaccine approach also consists of a similar procedure except for the in vitro transcription and other mRNA-related steps meaning that the manufacturing is simpler and time-saving [15]. Besides, DNA vaccines are characterized by a number of advantages over the conventional and even mRNA vaccines [41]. They are innately safe [42] as the vectors are not live, and hence cannot cause infection unlike vaccines based on viral vectors. DNA vaccines encode only the specific target antigen and are non-replicating [15]. No anti-vector immunity is induced after the immunization with DNA vaccines as the vector is not viral [15]. DNA vaccines are much more stable [43] as they exhibit stability at ambient temperatures, and do not have toxicity problems compared with mRNA vaccines [44,45]. DNA vaccine can be administered via intradermal, intramuscular, intravenous, subcutaneous, intranasal, and intranodal routes and manifest favorable immune response [41]. Additionally, DNA vaccines in clinical trials show a high safety profile [45,46]. A DNA vaccine is relatively inexpensive. Xia et al. have demonstrated that the handheld electroporation device with a microneedle electrode array can easily overcome the relative expensiveness of the conventional electroporation that is usually needed for inducing a strong immune response [16]. The development of DNA vaccines is time-saving as it is undemanding to produce large amounts of the target antigen compared with making proteins, growing viruses and bacteria, or synthesizing mRNA [45]. According to the recent pandemic experience, the speed of vaccine development is crucial especially when the infectious agent is characterized by a high mutation rate and high transmissibility. Thus, the most important requirements for the anti-COVID-19 vaccines that are safeness, protectiveness, accessibility, and inexpensiveness can be achieved via DNA vaccines [16]. Studies have already demonstrated the positive outcome of the DNA vaccine of SARS-CoV-2 spike protein [47,48]. Interestingly, there are already several DNA vaccines approved for veterinary use including a vaccine against highly pathogenic H5N1 Influenza A virus in chicken [49], the vaccine against West Nile virus in horses, etc. [50]. Most importantly, the very first DNA vaccine in humans that targets COVID-19 has recently obtained authorization for emergency use in India, 20 August 2021 [51]. Moreover, there is a number of DNA vaccines in phases 1, 2, or 3 of clinical development [51,52] while some are in the step of preclinical studies [41]. Most of the upcoming SARS-CoV-2 DNA vaccines are based on the whole S-protein [51]. Markedly, the advantageous outcome of DNA vaccines in preclinical development, that express the RBD of S-protein is also reported [53,54]. RBD plays a key role in the SARS-CoV-2 infection process [55] as it binds the ACE2 which is a primary receptor for the cellular entry of the virus [56,57,58]. Hence, blocking the interaction between RBD and ACE2 suggests that the antibodies induced via DNA-RBD immunization play a crucial role in the prevention of host infection. Additionally, a foldon tag that is fused with S1/S2 ectodomain is reported to be a good trimerization domain that successfully stabilizes the structures [23,24]. Besides, the addition of proline which was also used in this construct is found to stabilize the pre-fusion shape of the protein structure [19].

In the current study, the DNA vaccine “IgE-spike-S1/S2-D614G-6P-foldon” was designed and tested in vivo in BALB/c mice. The 3D structure of DNA vaccine encoded trimeric spike-ectodomain-D614G-6P antigen protein was determined using Swiss-Model homology modeling based on the template structure of the spike-2P (PDB ID: 6VSB) which shared a sequence identity larger than 99% with the target sequence. A comparison of the modeled structure with the spike-2P structure revealed that the additional four proline substitutions did not distort the S2 subunit conformation, consistent with previously reported data [59]. The modeled structure also indicated that the D614G mutation disrupts hydrogen bond formation with amino acid T859, promoting RBD in an “open” conformation, and increasing ACE2 receptor binding (Supplementary File S3). According to the validation cell experiment result, the DNA delivery was successfully enhanced via electroporation that was reflected in the expression of the antigen in HEK-293T cells. The three-dose regimen of immunization demonstrated that it can elicit antibody generation after the third booster dose. The three-dose regimen induced a high titer of antibodies in the sera of mice in all of the dose groups. Observed EPTs also make the overall picture rational. Moreover, the neutralization assay demonstrated that the sera of vaccinated mice could neutralize SARS-CoV-2 pseudovirus. However, the shortcomings of the study should be mentioned. The protective efficacy of the vaccine against SARS-CoV-2 pseudovirus was assessed in mice sera but the mice were not challenged with SARS-CoV-2 due to the absence of biosafety level 3 laboratory. Besides, although DNA vaccines are known to induce cellular immunity, T cell responses were not assessed in the current study. The immune response in male BALB/mice and other animal models such as non-human primates also need to be evaluated. This potentially limits the ability to generalize the outcome of the study. Despite the limitations that include the absence of a further evaluation of safety and efficacy, these data show that the DNA vaccine encoding IgE-spike-S1/S2-D614G-6P-foldon developed in this study appears to have a potentially protective effect on mice and deserves further assessment for optimization.

## 5. Conclusions

In summary, these initial results of the humoral immunogenicity induced via DNA encoding IgE-spike-S1/S2-D614G-6P-foldon immunization revealed that the vaccine candidate proposed in the current study seems to be a promising target for the future optimization and development. Hence, the further assessment of the proposed DNA construct as a potential vaccine candidate against COVID-19 is warranted.

## Data Availability

Not applicable.

## Supplementary Materials

The following supporting information can be downloaded at: https://www.mdpi.com/article/10.3390/v14051049/s1. Supplementary File S1. Curve data of EC50 values; Supplementary File S2. Complete sequence of DNA vaccine; Supplementary File S3. Structural characterization of DNA vaccine encoding trimeric spike-ectodomain-D614G-6P antigen protein.
