# Supplementary material for: Development of DNA Vaccine Candidate against SARS-CoV-2
Source: Viruses. 2022 May 15;14(5):1049. doi: 10.3390/v14051049 (PMC9144758; doi:10.3390/v14051049)
Supplement: Supplementary file 1 [file viruses-14-01049-s001.zip › Supplementary File 2.pdf]

Complete DNA plasmid sequence of "IgE-spike-S1/S2-D614G-6P-foldon":

GACTCTTCGCGATGTACGGGCCAGATATACGCGTTGACATTGATTATTGACTAGTTATTA
ATAGTAATCAATTACGGGGTCATTAGTTCATAGCCCATATATGGAGTTCCGCGTTACAT
AACTTACGGTAAATGGCCCGCCTGGCTGACCGCCCAACGACCCCCGCCCATTGACGTC
AATAATGACGTATGTTCCCATAGTAACGCCAATAGGGACTTTCCATTGACGTCAATGGG
TGGACTATTTACGGTAAACTGCCCACTTGGCAGTACATCAAGTGTATCATATGCCAAGT
ACGCCCCCTATTGACGTCAATGACGGTAAATGGCCCGCCTGGCATTATGCCCAGTACAT
GACCTTATGGGACTTTCCTACTTGGCAGTACATCTACGTATTAGTCATCGCTATTACCAT
GGTGATGCGGTTTTGGCAGTACATCAATGGGCGTGGATAGCGGTTTGACTCACGGGGAT
TTCCAAGTCTCCACCCCATTGACGTCAATGGGAGTTTGTTTTGGCACCAAAATCAACGG
GACTTTCCAAAATGTCGTAACAACTCCGCCCCATTGACGCAAATGGGCGGTAGGCGTGT
ACGGTGGGAGGTCTATATAAGCAGAGCTCTCTGGCTAACTAGAGAACCCACTGCTTACT
GGCTTATCGAAATTAATACGACTCACTATAGGGAGACCCAAGCTGGCTAGCGTTTAAAC
TTAAGCTTGCCACCATGGACTGGACCTGGATCCTGTTCCTGGTGGCCGCCGCCACAAGA
GTGCACAGCATGTTCGTGTTCCTGGTGCTGCTGCCCCTGGTGAGCTCTCAGTGCGTGAAC
CTGACCACAAGAACACAGCTGCCCCCCGCCTACACCAACAGCTTCACAAGAGGTGTCT
ACTACCCCGACAAGGTGTTCAGAAGCAGCGTCCTGCACAGCACCCAAGACCTGTTCCT
ACCATTCTTCAGCAACGTGACCTGGTTCCACGCCATCCACGTGAGCGGCACCAACGGCA
CCAAGAGATTCGACAACCCCGTGCTGCCCTTCAACGACGGCGTGTACTTCGCTAGCACC
GAGAAGAGCAACATCATCAGAGGCTGGATCTTCGGCACCACCCTGGATTCGAAGACAC
AGAGCCTGCTGATTGTGAACAACGCCACCAACGTGGTGATCAAGGTGTGCGAGTTTCA
GTTCTGCAACGACCCCTTCCTGGGGGTATACTACCACAAAAACAACAAGAGCTGGATG
GAGAGCGAGTTCAGAGTGTATTCGAGCGCCAACAACTGCACCTTCGAGTACGTGTCTCA
GCCCTTCTTGATGGACCTGGAGGGCAAGCAAGGCAATTTCAAGAATCTGAGAGAGTTC
GTGTTCAAGAACATCGACGGCTACTTCAAGATCTACAGCAAGCACACCCCCATCAACC
TGGTGAGAGACCTGCCCCAAGGCTTCAGCGCCCTGGAGCCCCTGGTGGACCTGCCCATC
GGCATCAACATCACAAGATTTCAGACCCTGCTGGCCCTGCACAGAAGCTATTTAACGCC
CGGCGACAGCAGCTCCGGATGGACCGCCGGGGCGGCAGCCTACTACGTGGGCTACCTG
CAGCCTAGAACCTTCCTGCTGAAGTACAACGAGAACGGCACCATAACGGACGCTGTTG
ACTGCGCCCTGGACCCCCTGAGCGAGACCAAGTGCACCCTGAAGAGCTTCACCGTGGA
GAAGGGCATCTATCAGACAAGCAACTTCAGAGTGCAGCCCACCGAGAGCATCGTGAGA
TTCCCCAACATCACCAACCTGTGCCCCTTCGGCGAGGTGTTCAACGCCACAAGATTCGC
TAGCGTGTACGCCTGGAATAGAAAAAGAATCAGCAACTGCGTGGCCGACTACAGCGTG
CTGTACAACAGCGCTAGCTTCAGCACCTTCAAGTGCTACGGTGTGAGCCCCACCAAGCT
GAACGACCTGTGCTTCACCAACGTGTACGCCGACAGCTTCGTGATCAGAGGCGACGAG
GTGAGACAGATCGCCCCCGGGCAGACCGGCAAGATCGCCGACTACAACTACAAGCTGC
CCGACGACTTCACCGGCTGCGTGATCGCGTGGAATAGCAATAACCTCGATAGCAAAGT
TGGCGGCAACTACAACTACCTGTACAGACTGTTCAGAAAGAGCAACCTGAAGCCCTTC
GAGAGAGACATCAGCACCGAGATCTACCAAGCCGGCAGCACCCCCTGCAACGGCGTG
GAGGGCTTCAACTGCTACTTCCCCCTGCAGAGCTACGGCTTTCAGCCCACCAACGGCGT

GGGCTATCAGCCCTACAGAGTGGTGGTGCTGAGCTTCGAGCTGCTGCACGCCCCCGCCA
CCGTGTGTGGCCCCAAGAAGAGCACCAACCTGGTGAAGAACAAGTGCGTGAACTTCAA
CTTTAACGGTCTGACCGGCACCGGCGTGCTGACCGAGAGCAACAAGAAGTTCCTGCCCT
TCCAACAGTTCGGCAGAGACATCGCCGACACCACCGACGCAGTGAGGGACCCTCAGAC
CCTGGAGATCCTGGACATCACCCCATGCTCATTTGGCGGCGTGAGCGTGATCACCCCCG
GCACCAACACAAGCAACCAAGTGGCCGTGCTGTACCAAGGCGTGAACTGCACCGAGGT
GCCCGTGGCCATCCACGCCGATCAGCTGACCCCCACCTGGAGAGTGTACAGTACCGGC
AGCAACGTGTTTCAGACAAGAGCCGGCTGCCTGATCGGCGCCGAGCACGTGAACAACA
GCTACGAGTGCGACATCCCCATCGGCGCCGGCATCTGCGCTAGCTATCAGACACAGAC
CAACAGCCCTAGAAGAGCTAGAAGCGTGGCTTCTCAGAGCATCATCGCCTACACCATG
AGCCTGGGCGCCGAGAACAGCGTGGCCTACAGCAACAACAGCATCGCCATCCCCACCA
ACTTCACCATCAGCGTGACCACCGAGATCCTACCTGTGTCAATGACCAAGACAAGCGT
GGACTGCACCATGTACATCTGCGGCGACAGCACCGAGTGCAGCAACCTGCTGCTGCAG
TACGGCAGCTTCTGCACACAGCTGAACAGAGCCCTGACCGGCATCGCCGTGGAGCAAG
ACAAGAACACCCAAGAGGTGTTCGCCCAAGTGAAGCAGATCTACAAGACCCCCCCCAT
CAAGGACTTCGGCGGCTTCAACTTCAGCCAAATCCTTCCCGACCCGAGCAAGCCTAGC
AAGAGAAGCCCCATCGAGGACCTGCTGTTCAACAAGGTGACCCTGGCCGACGCCGGCT
TCATCAAGCAGTACGGCGACTGCCTGGGTGACATCGCCGCTAGAGACCTGATCTGCGCT
CAGAAGTTCAACGGCTTAACCGTCCTCCCCCCCCTGCTGACCGACGAGATGATTGCTCA
GTACACTAGTGCATTGCTGGCCGGTACCATTACTTCCGGATGGACGTTTGGTGCGGGCC
CCGCCCTGCAGATCCCCTTCCCCATGCAGATGGCCTACAGATTCAACGGCATCGGCGTG
ACACAGAACGTGCTGTACGAGAATCAGAAGCTGATCGCCAATCAGTTCAACAGCGCCA
TCGGCAAGATCCAAGACAGCCTGAGCAGCACCCCTAGCGCCCTGGGCAAGCTGCAAGA
CGTGGTGAATCAGAACGCCCAAGCCCTGAACACCCTGGTGAAGCAGCTGAGCAGCAAC
TTCGGCGCCATCTCGAGCGTGCTAAACGACATCCTGAGCAGACTGGACCCCCCCGAGG
CCGAGGTGCAGATCGACAGACTGATCACCGGCAGACTGCAGAGCCTGCAGACCTACGT
GACACAGCAGCTGATCAGAGCCGCCGAGATCAGAGCTAGCGCCAACCTGGCCGCCACC
AAGATGAGCGAGTGCGTGCTGGGGCAGAGCAAGAGAGTGGACTTCTGCGGCAAGGGC
TACCACCTGATGAGCTTCCCTCAGAGCGCCCCCCACGGCGTGGTGTTCCTGCACGTGAC
CTACGTGCCCGCCCAAGAGAAGAACTTCACCACAGCCCCCGCGATCTGCCACGACGGC
AAGGCCCACTTCCCTAGAGAGGGCGTGTTCGTGAGCAACGGCACCCACTGGTTCGTGA
CACAGAGAAACTTCTACGAGCCTCAGATCATCACCACCGACAACACCTTCGTGAGCGG
CAACTGCGACGTGGTGATCGGCATTGTTAATAACACCGTGTACGACCCCCTGCAGCCCG
AGCTGGACAGCTTCAAGGAGGAGCTGGACAAGTATTTCAAGAACCACACAAGCCCCGA
CGTGGACCTGGGCGACATCAGCGGCATCAACGCTAGCGTGGTGAACATTCAGAAGGAG
ATCGACCGGCTGAATGAGGTGGCCAAGAACCTGAACGAGAGCCTGATCGACCTGCAAG
AGCTGGGCAAGTACGAGCAGTACATCAAGTGGCCCGGCTACATCCCCGAGGCCCCTAG
AGACGGCCAAGCCTACGTGAGAAAGGACGGCGAGTGGGTGCTGCTGAGCACCTTCCTG
CACCACCACCACCACCACTGAGAATTCTGCAGATATCCAGCACAGTGGCGGCCGCTCG
AGTCTAGAGGGCCCGTTTAAACCCGCTGATCAGCCTCGACTGTGCCTTCTAGTTGCCAG

CCATCTGTTGTTTGCCCCTCCCCCGTGCCTTCCTTGACCCTGGAAGGTGCCACTCCCACT
GTCCTTTCCTAATAAAATGAGGAAATTGCATCGCATTGTCTGAGTAGGTGTCATTCTATT
CTGGGGGGTGGGGTGGGGCAGGACAGCAAGGGGGAGGATTGGGAAGACAATAGCAGG
CATGCTGGGGATGCGGTGGGCTCTATGGCTTCTACTGGGCGGTTTTATGGACAGCAAGC
GAACCGGAATTGCCAGCTGGGGCGCCCTCTGGTAAGGTTGGGAAGCCCTGCAAAGTAA
ACTGGATGGCTTTCTCGCCGCCAAGGATCTGATGGCGCAGGGGATCAAGCTCTGATCAA
GAGACAGGATGAGGATCGTTTCGCATGATTGAACAAGATGGATTGCACGCAGGTTCTC
CGGCCGCTTGGGTGGAGAGGCTATTCGGCTATGACTGGGCACAACAGACAATCGGCTG
CTCTGATGCCGCCGTGTTCCGGCTGTCAGCGCAGGGGCGCCCGGTTCTTTTTGTCAAGAC
CGACCTGTCCGGTGCCCTGAATGAACTGCAAGACGAGGCAGCGCGGCTATCGTGGCTG
GCCACGACGGGCGTTCCTTGCGCAGCTGTGCTCGACGTTGTCACTGAAGCGGGAAGGG
ACTGGCTGCTATTGGGCGAAGTGCCGGGGCAGGATCTCCTGTCATCTCACCTTGCTCCTG
CCGAGAAAGTATCCATCATGGCTGATGCAATGCGGCGGCTGCATACGCTTGATCCGGCT
ACCTGCCCATTCGACCACCAAGCGAAACATCGCATCGAGCGAGCACGTACTCGGATGG
AAGCCGGTCTTGTCGATCAGGATGATCTGGACGAAGAGCATCAGGGGCTCGCGCCAGC
CGAACTGTTCGCCAGGCTCAAGGCGAGCATGCCCGACGGCGAGGATCTCGTCGTGACC
CATGGCGATGCCTGCTTGCCGAATATCATGGTGGAAAATGGCCGCTTTTCTGGATTCATC
GACTGTGGCCGGCTGGGTGTGGCGGACCGCTATCAGGACATAGCGTTGGCTACCCGTGA
TATTGCTGAAGAGCTTGGCGGCGAATGGGCTGACCGCTTCCTCGTGCTTTACGGTATCG
CCGCTCCCGATTCGCAGCGCATCGCCTTCTATCGCCTTCTTGACGAGTTCTTCTGAATTAT
TAACGCTTACAATTTCCTGATGCGGTATTTTCTCCTTACGCATCTGTGCGGTATTTCACAC
CGCATACAGGTGGCACTTTTCGGGGAAATGTGCGCGGAACCCCTATTTGTTTATTTTTCT
AAATACATTCAAATATGTATCCGCTCATGAGACAATAACCCTGATAAATGCTTCAATAA
TAGCACGTGCTAAAACTTCATTTTTAATTTAAAAGGATCTAGGTGAAGATCCTTTTTGAT
AATCTCATGACCAAAATCCCTTAACGTGAGTTTTCGTTCCACTGAGCGTCAGACCCCGT
AGAAAAGATCAAAGGATCTTCTTGAGATCCTTTTTTTCTGCGCGTAATCTGCTGCTTGCA
AACAAAAAAACCACCGCTACCAGCGGTGGTTTGTTTGCCGGATCAAGAGCTACCAACT
CTTTTTCCGAAGGTAACTGGCTTCAGCAGAGCGCAGATACCAAATACTGTCCTTCTAGT
GTAGCCGTAGTTAGGCCACCACTTCAAGAACTCTGTAGCACCGCCTACATACCTCGCTC
TGCTAATCCTGTTACCAGTGGCTGCTGCCAGTGGCGATAAGTCGTGTCTTACCGGGTTGG
ACTCAAGACGATAGTTACCGGATAAGGCGCAGCGGTCGGGCTGAACGGGGGGTTCGTG
CACACAGCCCAGCTTGGAGCGAACGACCTACACCGAACTGAGATACCTACAGCGTGAG
CTATGAGAAAGCGCCACGCTTCCCGAAGGGAGAAAGGCGGACAGGTATCCGGTAAGC
GGCAGGGTCGGAACAGGAGAGCGCACGAGGGAGCTTCCAGGGGGAAACGCCTGGTAT
CTTTATAGTCCTGTCGGGTTTCGCCACCTCTGACTTGAGCGTCGATTTTTGTGATGCTCGT
CAGGGGGGCGGAGCCTATGGAAAAACGCCAGCAACGCGGCCTTTTTACGGTTCCTGGG
CTTTTGCTGGCCTTTTGCTCACATGTTCTT
