# Supplementary material for: Development of DNA Vaccine Candidate against SARS-CoV-2
Source: Viruses. 2022 May 15;14(5):1049. doi: 10.3390/v14051049 (PMC9144758; doi:10.3390/v14051049)
Supplement: Supplementary file 1 [file viruses-14-01049-s001.zip › Supplementary File 3.pdf]

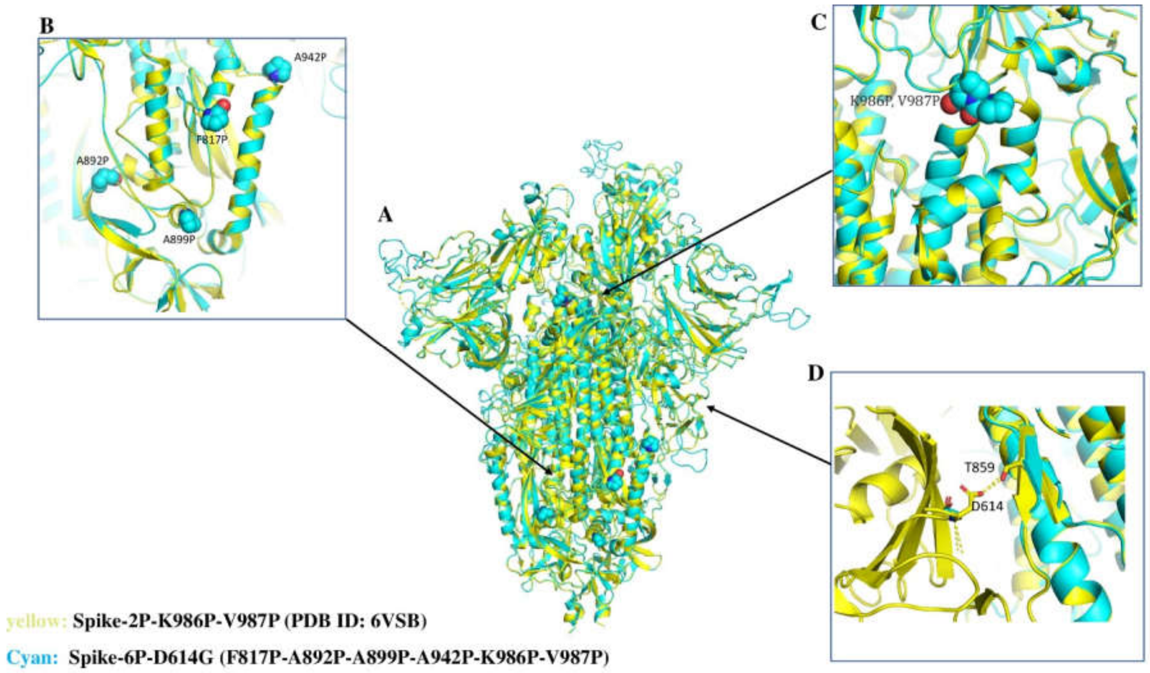

Structural characterization of DNA vaccine encoding trimeric spike-ectodomain-D614G-6P antigen protein.

(A) The alignment of modeled structure with the template structure of the Spike-2P (PDB ID: 6VSB). (B) Each of the four single point mutations introduced in S-6P compared with S-2P shown in detail. (C) The two single point mutations introduced in S-2P and S-6P shown in detail. (D) The hydrogen bond between amino acid D614 and T859.
